# Map and compass navigation: the mechanism and ontogeny of animal maps

**DOI:** 10.1016/j.anbehav.2025.123272

**Published:** 2025-09

**Authors:** Joe Morford, Joe Wynn, Patrick Lewin, Paris Jaggers, Lewis Lancaster-Reeves, Adelaide Sibeaux, Oliver Padget, Tim Guilford

**Affiliations:** a Department of Biology, University of Oxford, Oxford, U.K.; b Earth, Ocean and Ecological Sciences, University of Liverpool, Liverpool, U.K.; c Brain and Cognitive Sciences, University of Rochester, Rochester, NY, U.S.A.

**Keywords:** map and compass navigation, mechanism, ontogeny, path integration

## Abstract

Map and compass navigation, a two-step process in which animals use a map to determine goalward directions and a compass to orient in those directions, accounts for a variety of navigational behaviours across animals, from visual landmark navigation in familiar environments, to returning long distances from novel sites. However, while extensive investigation into the sensory basis of maps has implicated roles for visual, olfactory and magnetic cues, many details of their mechanism and ontogeny are less well understood. Here, we introduce a framework that deconstructs maps into three components: (1) cues, the nature of the environmental properties that animals use to determine goalward directions; (2) structure, the organization of information used to determine the goalward direction, distinguishing discrete and continuous map structures; and (3) implementation, encompassing strategies relating to how animals approach their goals and how animals combine multiple information sources. In some cases, inherited rules and imprinting are involved in the ontogeny of these components of map and compass navigation. However, in many instances, including those which require extrapolation of gradients or involve flexible navigation between multiple goals, extensive learning is probably required; none the less, how animals resolve the spatial arrangement of cues to learn maps, especially over large scales, is little understood. Mechanisms for determining vectors of self-motion, termed path integration, play an important role in map learning in mammals over relatively small spatial scales; we suggest that path integration could play a similar role in map learning in other taxa and over larger spatial scales. This would imply that path integration is more taxonomically widespread and plays a greater role in navigational learning than currently appreciated. This review helps to clarify links between disparate findings and raises questions about navigational mechanisms and ontogeny to better our understanding of map and compass navigation across taxa and scales.

## Highlights

• Map and compass navigation accounts for movement behaviours across taxa and scales.
• We consider three components of maps: cues, structure and implementation.
• We distinguish between maps with discrete and continuous structures.
• Path integration may play a role in map learning across diverse animal taxa.

**Figure 1:**
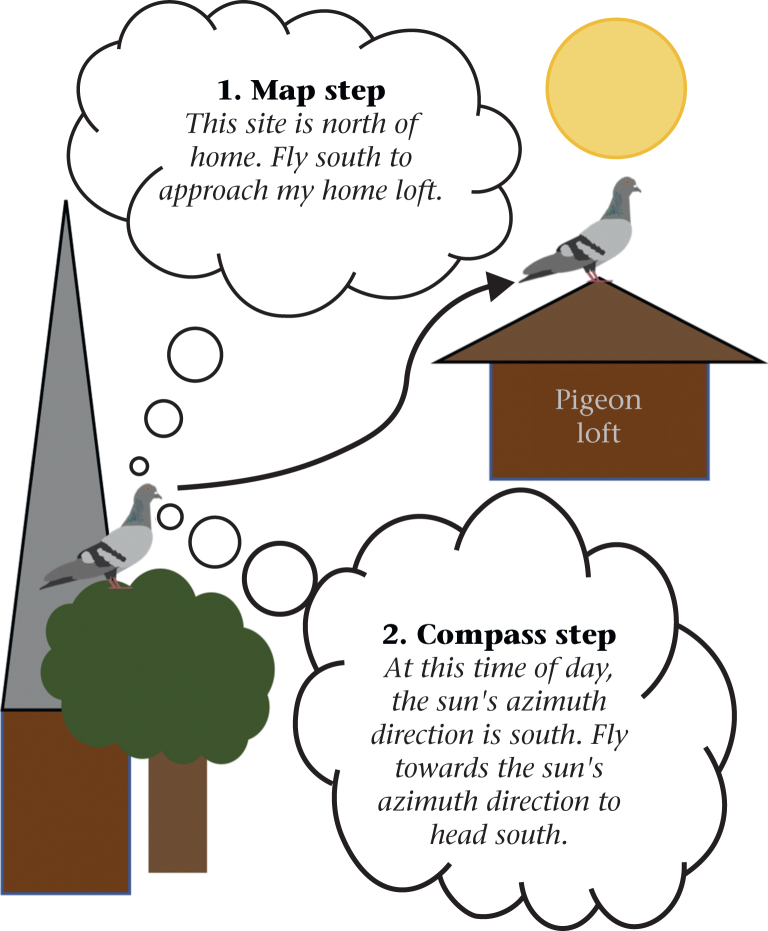
Map and compass navigation. A homing pigeon is shown using a two-step process to navigate to its home loft. The map step allows the pigeon to determine the compass direction of the goal, and the compass step allows it to move in that direction by relating the goalward compasses direction to its heading.

In the 1950s, Kramer introduced the idea that homing navigation involves a two-step mechanism, termed ‘map and compass’ navigation (Kramer, 1953). First, the map step involves determining an animal’s position in relation to ‘home’, other goals or other known parts of the environment. Most simply, this could comprise determining the direction of a single goal but, alternatively, might involve determining both the direction and distance to a goal or goals. Second, the compass step involves orienting in the direction of the goal by determining the relationship between the direction of the goal and egocentric heading. This conceptual framework for examining animal navigation is still central to research today (Gagliardo, 2013; Holland, 2014; Lohmann et al., 2022; R. Wiltschko & Wiltschko, 2003). Evidence supporting a two-step navigational process has accumulated through experiments that have independently manipulated animals' perception of map cues and compass cues (Baldaccini et al., 1975; Benvenuti & Wallraff, 1985; Chernetsov et al., 2017; Dall’Antonia et al., 1999; Ioalé et al., 1978; Kishkinev et al., 2021; Papi et al., 1974; Schmidt-Koenig, 1990; R. Wiltschko & Wiltschko, 1995), demonstrating that navigation, in these instances, involves a two-part mechanism: a map and a compass (Fig. 1).

Here, we focus on the mechanism and ontogeny of the map step of map and compass navigation. Tinbergen identified mechanism (‘causation’) and ‘ontogeny’ as two of the four major problems to be addressed by the research field of ethology, along with ‘survival value’ (adaptive value), and ‘evolution’ (Tinbergen, 1963). Much research on navigational maps in animals has focused upon the sensory basis of maps, while many of the details of their mechanism and ontogeny are less well understood (Wynn & Liedvogel, 2023). We introduce a framework on the mechanisms with which animals use maps in map and compass navigation with the aims of, first, clarifying the links between ideas and findings on animal maps across taxa and spatial scales and, second, generating questions on the less well studied and understood aspects of animal maps. This framework deconstructs animal maps into three interlinked components: (1) map cues, (2) map structure and (3) map implementation.

We then consider the ontogeny of animal maps, in particular in relation to the three components set out in our framework. Tinbergen gave particular emphasis to ontogeny and defined it as the ‘change of behaviour machinery during development’. He argued that addressing simple questions about how behaviour changes during development and what underlies these changes is the key to understanding this major problem for the field of ethology (Tinbergen, 1963). We address how map and compass navigation changes through development and discuss the roles of inherited rules and predispositions, imprinting and latent learning. Finally, we discuss mechanisms of map learning and consider the role of mechanisms for determining vectors of self-motion, termed path integration, in map learning across taxa and spatial scales.

## Map Mechanisms Framework

### Cues

Map cues comprise any environmental properties that an animal can perceive and use to determine goalward directions. In order to be useful for determining goalward direction, any such environmental property must be temporally stable and vary perceptibly and predictably in space. Extensive research on animal maps has focused upon the sensory basis of animal maps and, in particular, on map cues perceived through three sensory modalities: magnetic, visual and olfactory cues. Within each of these modalities, a range of cues are potentially available with various underlying spatial distributions. Several cues derived from the geomagnetic field have been implicated in providing animals with maps; these are magnetic inclination (K. Lohmann & Lohmann, 1994), magnetic intensity (K. J. Lohmann & Lohmann, 1996) and magnetic declination (Chernetsov et al., 2017; Packmor et al., 2024). Second, a large variety of visual cue types could provide animals with map information from landmark features (Collett et al., 1986) and their geometric relationships (Benhamou & Poucet, 1998; Cheng, 1986, 2005; Greene & Cook, 1997) to landscape features such as arrays of distant landmarks (Harten et al., 2020; Toledo et al., 2020) and prominent linear features (Biro et al., 2007; Kano et al., 2018; Lau et al., 2006). Third, there are a range of olfactants that animals might use to navigate with an olfactory map. However, while there is a large body of literature demonstrating the role of olfactory cues as a navigational map in homing pigeons (for reviews, see Gagliardo, 2013; Wallraff, 2004) and other birds (Benvenuti et al., 1993; Gagliardo et al., 2013; Holland et al., 2009; Padget et al., 2017; Pollonara et al., 2015; Wikelski et al., 2015), the identities and distributions of these olfactants have proved difficult to unravel. The limited exceptions include experiments in which animals have learned to navigate using introduced artificial odours in homing pigeons (Papi et al., 1974) and laboratory mice (Fischler-Ruiz et al., 2021). Some research has investigated the large-scale spatial distributions of putative olfactory map cues, indicating that, in certain regions, the ratios of olfactants or the concentrations of individual olfactants appear to be sufficiently temporally stable and spatially variable to be utilized as a map cue by animals (Wallraff, 2000, 2013; Wallraff & Andreae, 2000; Zannoni et al., 2020). None the less, linking experiments on olfactory navigation in animals to olfactant distributions remains the final step in unravelling the map cues that are the basis of navigation with an olfactory map. Finally, aside from the three aforementioned groups of map cues, various other sources of map cues have been suggested, including the sun arc (Matthews, 1951, 1953), the Coriolis force (Yeagley, 1947, 1951), infrasound (Hagstrum, 2000, 2013; Patrick et al., 2021) and gravity vectors (Blaser et al., 2014; Dornfeldt, 1991). However, there is currently relatively limited evidence in the form of controlled experiments demonstrating that these cues act as the basis of navigational maps in animals.

### Structure

**Figure 2:**
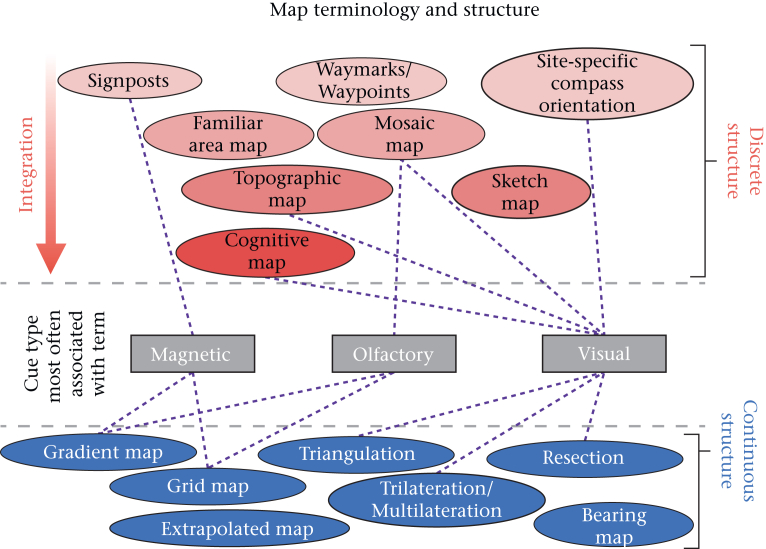
Map terminology and structure. We have grouped the different terms for animal maps into those that describe maps with discrete structure (red, top section) and with continuous structure (blue, bottom section). Terms used to describe maps with a discrete structure are placed and shaded according to their integration of directional information in the described maps: e.g. the term ‘cognitive map’ typically describes fully integrated maps encoding all of the spatial relationships between recognized places, whereas the term ‘signpost’ describes a map encoding single goalward directions from each site. Various terms have predominantly been used in reference to map cues in a particular sensory modality; in these cases, we have connected terms with dotted lines to the cue type or types with which they are most often associated in the literature.

Map structure is the organization of information used to determine the direction of goals. We distinguish between maps with discrete and continuous structures, as shown in Fig. 2. Maps with discrete structures, in which different places or regions are recognized and function distinctly in providing animals with directions to goals, have been invoked using a large number of different terms, sometimes with reference to maps based on cues in a particular sensory modality. These include mosaic maps (Papi et al., 1972; Wallraff, 1974), topographic maps (Wallraff, 1988), familiar area maps (Baker, 1978), cognitive maps (O’Keefe & Nadel, 1978; Tolman, 1948), sketch maps (Jacobs & Schenk, 2003), signposts (T. S. Collett & Collett, 2011) and site-specific compass orientation (Gagliardo et al., 1999; Luschi & Dall’Antonia, 1993). While these all comprise maps with discrete structures, they can none the less differ in how they organize spatial information. At one end of the spectrum, discrete maps can be structured such that they provide animals with only the directions to single goals from each distinctly recognized place. This could facilitate moving along the next step of a migratory trajectory (e.g. in juvenile loggerhead turtles utilizing magnetic signposts; T. S. Collett & Collett, 2011; Lohmann et al., 2001; Lohmann et al., 2012), or return to a single goal; for example, the home loft of homing pigeons or the breeding island of pelagic seabirds engaged in central place foraging. At the other end of the spectrum, a map with discrete structure might be fully integrated, encoding all of the spatial relationships between each of the recognized places in the map. This would potentially facilitate novel shortcutting between different places and is the most common conception of mammalian cognitive maps (O’Keefe & Nadel, 1978). More generally, maps with discrete structures can differ in the density and integration of their organization of directional information, but all involve distinct recognition and orientation in response to different places or regions in space.

By contrast, maps with continuous structures allow animals to exploit predictable spatial patterns in environmental cues to obtain continuous map information. This allows them to determine their direction to goals flexibly through space, rather than recognizing discrete regions. There is the potential for magnetic and olfactory cues, among other cues that are distributed along spatial gradients in the environment, to be utilized by animals as maps with continuous structure, termed grid maps, gradient maps or bearing maps (Gould, 1982; Jacobs & Schenk, 2003; Papi, 1990; Putman et al., 2011; Wallraff, 1974, 2004; Yeagley & Whitmore, 1947). Predictable variation in the spatial arrangement of familiar visual landmarks could also provide animals with continuous spatial information. For instance, taking back-bearings from two or more visual landmarks of known position can reveal a navigator’s location at their point of intersection and hence facilitate determination of goalward directions at novel locations. This is termed resection by intersection (Johnson, 1970; Mooers, 1972), and similar mechanisms include utilizing triangular geometry between a novel site and two known sites (triangulation/self-triangulation, Baker, 1984; Johnson, 1970) and utilizing the distances to known sites to determine position (trilateration/multilateration). The involvement of these mechanisms in map and compass navigation has been suggested to explain the partial effect of experimental manipulations on time-compensated sun compass use in birds (Padget et al., 2018). Egyptian fruit bats appear to use the spatial arrangement of familiar visual landmarks to navigate from novel sites; this has been described as self-triangulation and as cognitive map navigation (Harten et al., 2020; Toledo et al., 2020; Tsoar et al., 2011). None the less, the term cognitive map has been mainly used elsewhere to describe maps with a fully integrated discrete structure (as detailed above) rather than maps with continuous structure facilitating goalward navigation from novel sites through triangulation or other similar mechanisms.

**Figure 3:**
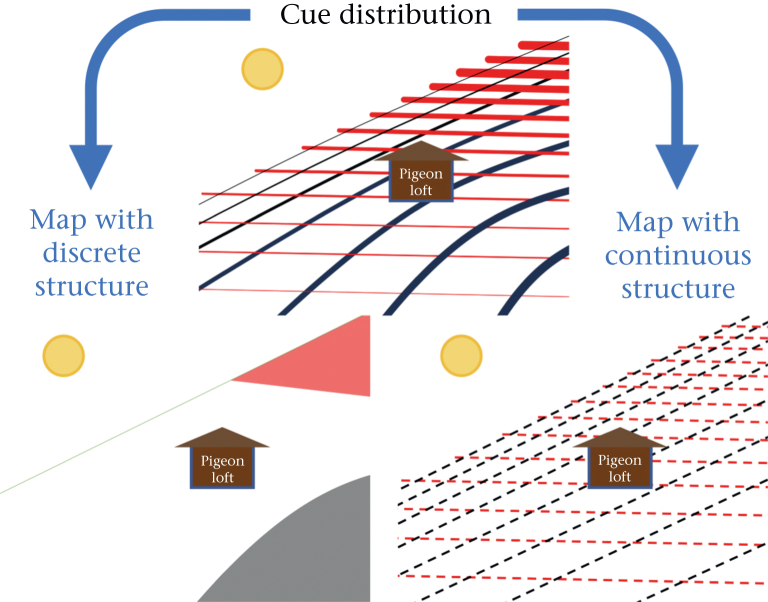
Cue distribution and map structure. An environment with two cues distributed along gradients (red and black solid lines) is shown in the top panel. The bottom panels show two ways in which a map of this environment could be structured. On the left, a representation of a map with a discrete structure is shown, comprising two regions, one with high values of the red cue and one with high values of the black cue, that can be recognized and function distinctly in providing animals with a goalward direction to the home loft. On the right, a representation of a map with a continuous structure is shown, with a linear approximation of the two gradient fields facilitating approximate goalward orientation towards the pigeon loft continuously through the environment.

Exploiting predictable variation in the spatial distributions of cues could potentially facilitate ‘true navigation’, the ability to navigate to a known goal after displacement to novel far-off sites without direct sensory contact with the goal, and ‘migratory true navigation’, the ability to correct or compensate for displacements from a migratory trajectory (for reviews, see Holland, 2014; Phillips et al., 2006). How the predictable variation in the spatial distribution of cues is organized in an animal’s map will impact upon the accuracy and efficiency of the animal’s navigation. Environmental gradients are often noisy and do not necessarily vary in simple linear and orthogonal patterns (Boström et al., 2012; Wallraff & Andreae, 2000; Wynn, Padget, Morford, et al., 2022; Zannoni et al., 2020; Åkesson & Alerstam, 1998). It is therefore possible that animals might approximate spatial gradients, potentially as varying linearly and/or orthogonally in space, as illustrated in Fig. 3. This might generate predictable patterns in orientation error in animal movements, which could allow us to assess the way in which animal maps are structured (Turner et al., 2016).

The structure of information used by animals to determine a goalward direction is somewhat limited by the underlying spatial distribution of map cues. For instance, if it smells of olive oil north of the goal and turpentine south of the goal, without any further variation in the distribution of these olfactants, the organization of information that the animal could use in its navigational map is limited to comprising a simple discrete structure by this underlying olfactant distribution. However, the structure of an animal’s map is not entirely determined by the underlying spatial distribution of map cues but also depends on the way in which the animal perceives and orients in response to the cue. Even if a cue is distributed along gradients in space, an animal might perceive and/or orient in response to discrete ranges of the cue’s value. This animal could be said to have a map with a discrete structure; this is illustrated in Fig. 3. In some regions magnetic inclination varies with latitude (Boström et al., 2012; Wynn, Padget, Morford, et al., 2022; Åkesson & Alerstam, 1998) and, for instance, forms a relatively predictable and stable gradient over the migratory trajectory of loggerhead sea turtles circumnavigating the North Atlantic gyre (Lohmann et al., 2001, 2012). None the less, if these hatchling turtles only recognize and respond to the magnetic coordinates of specific, distinct regions of the migratory trajectory, the structure of their map does not constitute a continuous gradient or grid, but a discrete structure, of a limited set of regions or coordinates (T. S. Collett & Collett, 2011; Lohmann et al., 2001, 2012; Putman, Scanlan, et al., 2014). Similarly, if an animal perceives distinct patches of olfactants in different regions, and uses these to orient towards goals, we can consider the structure of this olfactory map to have a discrete rather than continuous structure, even if the olfactants are distributed along gradients in space (Gagliardo, 2013). It therefore currently remains unclear whether the olfactory maps of homing pigeons and some seabirds are organized with a discrete or continuous structure, despite air sampling studies examining the spatial distributions of olfactants through space (Wallraff, 2000, 2013; Wallraff & Andreae, 2000; Zannoni et al., 2020). However, correlative analysis of the homing trajectories of Manx shearwaters have shown that they orient on the goalward beeline direction despite intervening obstacles, and that departure time from distant foraging sites is predicted by the goalward beeline distance; these results appear consistent with a map with continuous structure encoding the direction and distance to their breeding colony (Padget et al., 2019). Further experimental and correlative studies that aim to determine how animals organize spatial information and utilize high-resolution animal tracking data are required to better understand the structure of animal maps.

### Map Implementation

There are various implementations of navigational maps that animals could employ to move goalward. These encompass strategies relating to both how animals approach their goals and how animals combine multiple map cues to determine goalward directions. For instance, animals with discrete map structures might approach their goal by navigating along a sequence of waypoints/waymarks, orienting at each waypoint towards the next (Lohmann et al., 2022). This is consistent with the orientation responses of hatchling loggerhead turtles in response to recognized regions on their migratory trajectory (Lohmann et al., 2001, 2012). Additionally, this could be one of the mechanisms involved in homing pigeon navigation along familiar, stereotyped and sometimes relatively inefficient routes (Biro et al., 2004; Meade et al., 2005), in which map and compass navigation between waypoints on a route may be combined with other route-following mechanisms to provide navigational guidance (Biro et al., 2007). Alternatively, animals could orient directly towards their goal using their navigational map; in some circumstances, this might represent a more efficient navigational strategy. This appears to be employed by Manx shearwaters in their homing flights even from far-off sites and despite the presence of intervening obstacles (Padget et al., 2019).

In scenarios in which multiple map cues are available, animals might implement various strategies to combine these and orient in goalward directions. Various examples of map and compass navigation have been shown to involve multiple map cues: visual and olfactory map cues in homing pigeon and Scopoli’s shearwater navigation (Gagliardo, Odetti, & Ioalè, 2001; Padget et al., 2017; Pollonara et al., 2015); magnetic inclination and intensity in migratory navigation of hatchling turtles (Lohmann & Lohmann, 1994, 1996; Putman et al., 2011); magnetic inclination and declination in reed warbler migratory navigation (Packmor et al., 2024). When multiple different map cues are utilized by animals simultaneously, we can ask how animals combine these cues to determine goalward directions. Does one source of map information take precedence over others? Are multiple cues used together to disambiguate location? How do animals behave if different map cues conflict? Experiments putting featural landmark and geometric information into conflict in rats have revealed that they will learn both sources of map information but that geometric information takes precedence over featural information when the two are put into conflict (Batty et al., 2009; Benhamou & Poucet, 1998; Wall et al., 2004). Over larger spatial scales, in one study, reed warblers appeared to ignore changes in magnetic map cues if the cues were presented in unrealistic combinations (Kishkinev et al., 2021). These studies reveal how the combination of map cues can be important in determining the orientation responses of animals.

Various implementation strategies for navigation using cues distributed along spatial gradients have been proposed. For instance, it has been suggested that animals might travel along map isolines or coastlines to find a goal located at a known value of a single gradient cue (Lohmann et al., 2022; K. J. Lohmann & Lohmann, 2006). Similarly, on return migration, Eurasian reed warblers appear to orient in a particular compass direction until they intercept a known value of magnetic inclination (Wynn, Padget, Mouritsen, et al., 2022). Additionally, for navigating with multiple gradients, a simple strategy of approaching the goal in each gradient alternately has been suggested (Lohmann et al., 2007, 2022). However, combining positional information from multiple gradients could facilitate more efficient goalward navigation. In the case of grid maps of two orthogonally varying gradients, it seems intuitive that the vectors of displacement in each gradient field could be summed to provide the homeward vector. However, in cases where animals may use grid maps comprising nonorthogonal and/or nonlinearly varying gradients, combining the gradients to determine the homeward vector is likely to be more complex. Various models of how animals might combine multiple gradients, in particular when they vary nonorthogonally and nonlinearly, have been suggested in theoretical models of grid map navigation (Benhamou, 2003; Turner et al., 2016). Different models vary in the apparent simplicity of how they combine gradients, as well as in the assumed structure of underlying gradients. Contemporary developments in animal tracking technology (Beardsworth et al., 2022; Couzin & Heins, 2022) may allow spatial patterns in navigational performance to be quantified so that the predictions of these models can be tested. Further cue conflict and combination experiments could also provide better understanding of how different sources of map information are combined.

## The Ontogeny of Maps

### The Ontogeny of Map Cues

In considering the ontogeny of the cues utilized in animal maps, various questions arise. What are the roles of inherited rules and learning in determining what map cues animals utilize? How do the cues they utilize depend upon their developmental environment? How do the cues they utilize change through development?

The cues that animals use as a map could rely upon inherited rules and even be developmentally fixed. For example, hatchling loggerhead turtles and juvenile salmon appear to inherit rules for orientation in response to certain magnetic cues: magnetic inclination and magnetic intensity (Lohmann et al., 2001, 2012; Lohmann & Lohmann, 1994, 1996; Minkoff et al., 2020; Putman, 2015; Putman et al., 2011, 2015, 2020; Putman, Scanlan, et al., 2014; Scanlan et al., 2018). Some systems largely controlled through inherited rules might also involve some learning: imprinting upon the magnetic cues at the natal site, for instance, involves an inherited predisposition accompanied by rapid, relatively fixed learning. Examples include the imprinting of loggerhead sea turtles, salmon, Manx shearwaters and Eurasian reed warblers upon the olfactory or magnetic cues at their natal site (Brothers & Lohmann, 2015, 2018; K. J. Lohmann & Lohmann, 2019; Putman et al., 2013; Putman, Jenkins, et al., 2014; Scholz et al., 1976; Wynn et al., 2020; Wynn, Padget, Mouritsen, et al., 2022). In cases of imprinting, learning is likely to be extremely constrained, with animals predisposed to use particular cues and learning therefore restricted to variation in the values of those cues.

Other more complex learning programs might also be relatively constrained in which cues can be learned and when learning can occur, or could be less constrained, facilitating learning map cues flexibly depending upon the developmental environment. Homing pigeons have been shown to have a sensitive learning period for developing an olfactory map using wind blowing through their home loft in the 3–4 months after fledging (Gagliardo, Ioalè, et al., 2001). During this period, they associate changes in olfactants with the wind direction (Gagliardo, 2013; Gagliardo, Ioalè, et al., 2001; Odetti et al., 2003). This mechanism appears to involve the latent learning of olfactory cues while confined to their home loft, such that olfactory map cues are learned before they are utilized for navigation. It is therefore likely that inherited predispositions and constraints determine the cues that are latently learned during this sensitive learning period. The nature of these inherited predispositions is not yet clear: hypothetically, pigeons could learn any olfactant or ratio of olfactants that changes with wind direction; conversely, they might be primed to learn specific olfactants. However, homing pigeons can also learn to use map cues outside of this sensitive learning period. Older homing pigeons can learn olfactory and visual map cues when free flying, rather than confined to the loft, outside of the sensitive early learning period (Odetti et al., 2003). Learning map cues perceived through multiple sensory modalities and outside of the sensitive learning period may be less constrained by inherited predispositions. Moreover, if animals are flexible in what map cues they learn, depending on which are most pertinent in their environment, this might explain the range of different cues implicated in homing pigeon navigation in different experiments and at different locations (Wiltschko et al., 1987).

Visual landscapes represent an example in which the specific map cues used during navigation seem less likely to be developmentally fixed, with the spatial and temporal change in visual landscapes potentially preventing successive generations of animals from using the same visual map cues. Discrete map structures of visual cues must therefore be learned by animals as they obtain experience of their environment. However, these cues might still be constrained in various ways; for instance, in regard to the size, distance or geometric features of the visual cues used.

### The Ontogeny of Map Structure

How animals organize map information may also change through development and involve inherited constraints and predispositions as well as learning. In some cases, the organization of map information appears to rely on inherited rules, such as the discrete map structure of sea turtles and salmon used to orient in specific compass directions on their migratory trajectory (Lohmann et al., 2001; Lohmann & Lohmann, 1994, 1996; Minkoff et al., 2020; Putman, 2015; Putman et al., 2011, 2015, 2020; Putman, Scanlan, et al., 2014; Scanlan et al., 2018). Whether the structure of the magnetic map utilized by turtles and salmon remains developmentally fixed, or changes through learning, is less well understood. The use of magnetic cues in the homing navigation of green turtles displaced up to 100 km from their nesting islands (Luschi et al., 2007) appears indicative of a more complex map structure than a small number of inherited signposts. This suggests that a simple, discrete inherited map structure may have been extended and developed through learning. The number of discrete regions that the turtles recognize and orient in response to might have increased through development. Further, it is possible that these animals have developed the ability to exploit the gradient structure of the magnetic field and utilize a continuous magnetic map through learning.

One suggestion regarding the ontogeny of magnetic map structures is that an inherited grid map of magnetic gradients could be calibrated by the magnetic coordinates of the natal site (Putman, 2015). This would represent a developmentally fixed learning event, analogous to imprinting, in combination with relatively complex inherited rules, and is consistent with some experimental work on salmon (Putman, Jenkins, et al., 2014). The advantage of a system of calibration would be that it could alleviate one of the problems of inheriting a complex magnetic map: secular variation, the way in which the magnetic field varies through time (Wynn, Padget, Morford, et al., 2022). Another possible interplay of inheritance and learning in the ontogeny of map structure could involve the parameterization of a relatively constrained map structure. For instance, it has been suggested that animals might use maps of one latitudinal and one longitudinal cue, forming an orthogonal grid map (Gould, 2008; Lohmann et al., 2007; Putman et al., 2011). This could represent a constrained structure that animals parameterize through learning map cues that vary with latitude and longitude. Alternatively, some animal map structures might be relatively unconstrained by inherited predispositions and instead be flexible and dependent on the structure of the available cues within the developmental environment of animals. Learning map structures probably involves determining how cues vary locally, and this might then facilitate extrapolation to areas beyond an animal’s range of experience (Kishkinev et al., 2021; Turner et al., 2016), potentially through generalization of associations learned through local, familiar area navigation (Guilford & Burt de Perera, 2017). Hence, learning probably plays an important role in the ontogeny of relatively more complex map structures in certain systems, but inherited predispositions to learn maps with certain structures and to approximate and extrapolate cue distributions in certain ways might also be crucial.

### The Ontogeny of Map Implementation

The ways in which animals develop map implementation strategies remain relatively unexplored. Some strategies, such as loggerhead turtles migrating by following a series of waypoints, may rely upon inherited rules, at least on first migration (Lohmann et al., 2001, 2012). However, the roles of inherited rules in following map isolines or coastlines and the ways in which these strategies change through development as animals learn are unknown. Similarly, rules for combining map cues, such as responding to different map cues independently, prioritizing some map cues over others, or only responding to realistic combinations of map cues, might change as animals develop and learn or comprise inherited, fixed rules. Homing pigeons trained to find hidden food in a rectangular environment relied upon featural landmark and geometric spatial information to differing extents depending upon their training environment (Kelly et al., 1998). This demonstrates how rules for combining spatial information can be influenced through learning and depend upon the developmental environment of animals. However, the ontogenies of rules for combining discretely structured map cues for map and compass navigation over larger spatial scales in animals are little known.

Similarly, mechanisms of combining continuously structured map cues to determine the direction of goals could be fixed, inherited systems, or might emerge through learning. While various theoretical models of gradient combination in grid map navigation, associated with specific predictions on the spatial distribution of orientation errors, have been postulated (Benhamou, 2003; Turner et al., 2016), their ontogeny has been little considered. These questions may appear difficult to investigate; however, contemporary developments in animal tracking technology provide the potential to test the predictions of navigational models, and quantify navigational performance not only in relation to its spatial distribution, but also its changes through development. This has the potential to better our understanding of the ontogeny of navigational strategies in animal maps, as well as the ontogeny of map cues and structure.

### Map Learning and the Role of Path Integration

As set out above, learning probably plays an important role in the ontogeny of animal maps in many examples of map and compass navigation. During map learning, animals need to resolve the spatial arrangement of map cues. However, beyond the simplest instances involving imprinting on the map cues at the natal site, there are few systems in which map learning is well understood. When learning cognitive maps in new environments over relatively small spatial scales, mammals may associate map cues with vectors obtained during exploration through tracking their own travel. By integrating distance and direction information, they are able to compute a ‘home’ vector that takes them back to a familiar reference point or ‘home base’ by the straightest possible path (Etienne & Jeffery, 2004; Fischler-Ruiz et al., 2021; McNaughton et al., 1996, 2006; Savelli & Knierim, 2019; Wang, 2016; Whittington et al., 2022). This vector allows animals to determine the distance and direction between sites of interest. Mechanisms of tracking vectors of self-motion are termed ‘path integration’ or ‘dead reckoning’ (Mittelstaedt & Mittelstaedt, 1980), and involve utilizing compass or idiothetic (angular self-motion) directional cues in combination with speed or distance estimation through mechanisms such as stride integration and optic flow integration (Cheung et al., 2007, 2008; Vickerstaff & Cheung, 2010; Wittlinger et al., 2007). Therefore, path integration has the potential to provide an animal accurate distance and direction information between discrete map cues or sites during the map-learning process, at least for small-scale navigation. Similarly, it has been suggested that bees and ants might associate snapshot views with path-integrated vectors to learn a map, although clear evidence that this occurs is lacking (M. Collett & Collett, 2000; Müller & Wehner, 2010; Wehner, 2008).

However, it is unclear whether similar learning mechanisms involving path integration could scale up to facilitate map learning in animals performing map and compass navigation over larger spatial scales. First, while path integration has been found in a wide range of taxa, including a range of insects (Chittka et al., 1995; Müller & Wehner, 1988; Neuser et al., 2008), spiders (Moller & Görner, 1994), crabs (Layne et al., 2003; Zeil, 1998), birds (von Saint Paul, 1982), fish (Sibeaux et al., 2024), rodents (Etienne et al., 1988; Mittelstaedt & Mittelstaedt, 1980) and dogs (Séguinot et al., 1998), it has been little studied in taxa that exhibit map and compass navigation over long distances. Further, the only study finding evidence of path integration in birds was conducted in geese that were walking, rather than flying, to their goal (von Saint Paul, 1982). Second, learning a map through mechanisms involving self-motion cues would require path integration that is not overly susceptible to noise over the spatial scales at which map cues vary; it is unclear whether this is possible for instances of map and compass navigation over large spatial scales.

**Figure 4:**
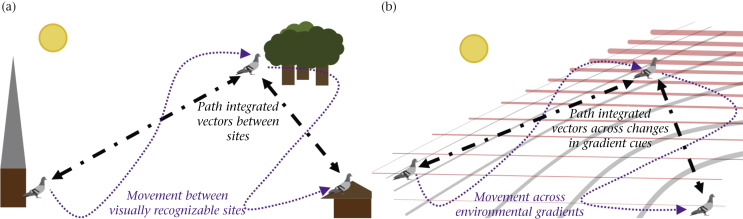
Map learning and path integration. Map learning may involve the integration of changes in map cues with vectors of self-motion as animals explore their environment. Panel (a) shows how learning a map with discrete structure could involve an association between the visual map cues at a site and a path-integrated vector between sites. Similarly, panel (b) shows how learning continuous map structures could involve the integration of changes in map cues, such as magnetic or olfactory gradients, with path-integrated vectors, to enable learning of how map cues vary through space.

One map-learning mechanism that does not appear to involve path integration is the mechanism of learning an olfactory map in homing pigeons, in which pigeons associate the direction of the wind with detected olfactants in their home loft (Gagliardo, 2013; Gagliardo, Ioalè, et al., 2001; Odetti et al., 2003). This allows homing pigeons to somewhat resolve the spatial arrangement of map cues without themselves moving. However, even if learning from wind-driven olfactants allows individuals to resolve the spatial arrangement of olfactory cues relative to their home loft (e.g. it smells of turpentine in the south), it remains unclear how this process could facilitate certain map navigational abilities such as determining the distance to navigational goals (e.g. Padget et al., 2019), or navigating to multiple targets (e.g. Blaser et al., 2013). Further, in many other examples of map and compass navigation, a mechanism of learning maps while confined to a single site seems unlikely or even impossible. Animals must move to experience how certain map cues vary in space; these include magnetic cues and visual cues beyond an animal’s visual field. Indeed, this may also be the case for odour cues in many environments, in which animals are not exposed to the wind sufficiently to learn a map, or in which the winds do not provide sufficient information about how odours vary in space. It is therefore likely that, in some cases, the ontogeny of maps may involve learning during movement, by tracking how cues vary through space. This probably necessitates the integration of changes in map cues with vectors of self-motion (Fig. 4), despite the limited evidence currently available to support the idea that path integration takes place in the relevant taxa and across sufficient spatial scales.

With path integration, learning discrete map structures, including site-specific compass orientations and mosaic olfactory maps, could involve an association between map cues at a site and a self-motion vector between sites. Learning continuous map structures could involve the integration of changes in map cues, such as magnetic or olfactory gradient values, with path-integrated vectors. Integrating self-motion vectors with changes in map cues would potentially enable learning how different cues vary through space and facilitate development in the structuring of map information and navigational strategy through learning. This mechanism of learning may play a role in the ontogeny of animal maps, and it is unclear how maps could be learned without some way of tracking self-motion, with the exception of learning odours carried on the wind. We suggest that future studies could examine whether path integration occurs in relevant taxa and over the spatial scales required for learning a large-scale navigational map; these could include experimental studies of path integration using traditional designs, as well as studies of orientation error in high resolution tracks of free-ranging animals to assess whether these are consistent with models of path integration.

## General Conclusions

There are many outstanding questions relating to the mechanisms and ontogeny of the map step of map and compass navigation in animals. We have set out a framework that deconstructs animal maps into three interlinked components: map cues, map structure and map implementation. The sensory basis of animal maps has been extensively researched, and hence the cues that underly animal maps are the best understood of these three components. We set out a classification of map structures that distinguishes discrete and continuous map structures and helps to clarify the links between findings on the organization of spatial information in different taxa and across various spatial scales. Our understanding of the map implementation strategies employed by animals, beyond theoretical ideas and models, is currently relatively limited. These strategies include the way in which animals approach their goals and their mechanisms of combining multiple map cues. There is a role for inherited rules and imprinting in the ontogeny of maps in some animals, but the roles of learning predispositions and the mechanisms of more complex learning programs still require investigation. Path integration plays an important role in the development of cognitive maps in mammals, but it is unclear how map learning occurs in other taxa and across larger spatial scales. We suggest that path integration might play an unappreciated role in map learning in some animals using map and compass navigation by allowing animals to integrate self-motion vectors with changes in map cues as they move and learn even across large spatial scales. This would imply that path integration is more widespread, taxonomically and in context, than currently appreciated. We suggest that future studies could examine whether path integration occurs in relevant taxa and over the spatial scales required for learning a large-scale navigational map. Finally, we expect that contemporary advances in the availability of large tracking data sets of animal movements will allow us to test the assumptions and predictions of navigational models, to shed light upon the navigational mechanisms that animals employ, in particular the way in which animals structure spatial information and employ navigational strategies during movement, and how these change through development. This has the potential to better our understanding of the mechanisms of animal maps, and their ontogeny, across a range of systems and spatial scales.

## Author Contributions

**Joe Morford:** Writing – review & editing, Visualization, Writing – original draft, Conceptualization. **Joe Wynn:** Writing – review & editing, Conceptualization. **Patrick Lewin:** Writing – review & editing, Conceptualization. **Paris Jaggers:** Writing – review & editing, Conceptualization. **Lewis Lancaster-Reeves:** Writing – review & editing, Conceptualization. **Adelaide Sibeaux:** Writing – review & editing. **Oliver Padget:** Conceptualization, Supervision. **Tim Guilford:** Writing – review & editing, Conceptualization, Supervision.

## Declaration of Interest

The authors have no competing interests to declare relevant to this article’s content.
